# Supplementary material for: Identification of Biological Functions and Prognostic Value of NNMT in Oral Squamous Cell Carcinoma
Source: Biomolecules. 2022 Oct 15;12(10):1487. doi: 10.3390/biom12101487 (PMC9599733; doi:10.3390/biom12101487)
Supplement: Supplementary file 1 [file biomolecules-12-01487-s001.zip › biomolecules-1883645-supplementary.pdf]

Supplementary Figure. We first performed transient transfection of OSCC3 cells for 24, 36 and 48 hours using small interfering RNA kits (siRNA, Guangzhou RiboBio, china) for NNMT, including three siRNAs and control. And we detected their NNMT mRNA expression after transfection by PCR respectively, so as to screen the base sequence and transfection time with the highest transfection efficiency. After that, the siRNA2 with the highest transfection efficiency was cloned into pLVX-Puro, i.e. pLVX-shNNMT. The full length of the cDNA sequences of NNMT was cloned into lentivirus vector pCDH-CMV, i.e. pCDH-NNMT. Plasmids were transfected into 293T cells via Polyjet and the superna-tants were collected 2 days after transfection. After being filtered through a 0.45-μm PES filter, the viral supernatants were either directly added to OSCC3 or stored at -80 °C.

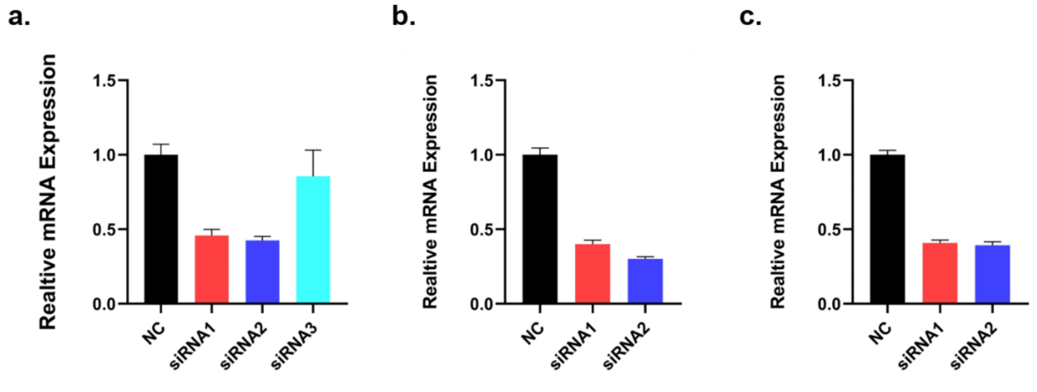

Figure S1. Exploration of optimal transfection conditions with small interfering RNA. (a) Relative mRNA expression levels of NNMT after transient transfection with three siRNAs for 24 hours. (b) Relative mRNA expression levels of NNMT after transient transfection with siRNA1 and siRNA2 for 36 hours. (c) Relative mRNA expression levels of NNMT after transient transfection with siRNA1 and siRNA2 for 48 hours. siRNA, small interfering RNA.
